# Inguinal or inguino-iliac/obturator lymph node dissection after positive inguinal sentinel lymph node in patients with cutaneous melanoma

**DOI:** 10.2478/v10019-012-0041-z

**Published:** 2012-07-24

**Authors:** Nebojsa Glumac, Marko Hocevar, Vesna Zadnik, Marko Snoj

**Affiliations:** 1 Department of Surgical Oncology, Institute of Oncology, Ljubljana, Slovenia; 2 Cancer Registry, Institute of Oncology, Ljubljana, Slovenia

**Keywords:** melanoma, inguinal, sentinel lymph node biopsy, micrometastases, lymphadenectomy

## Abstract

**Background:**

The aim of the study was to determine whether the presence of inguinal sentinel lymph node (SLN) metastases smaller than 2 mm (micrometastases) subdivided according to the number of micrometastases predicts additional, non-sentinel inguinal, iliac or obturator lymph node involvement in completion lymph node dissection (CLND).

**Patients and methods.:**

Positive inguinal SLN was detected in 58 patients (32 female, 26 male, median age 55 years) from 743 consecutive and prospectively enrolled patients with primary cutaneous melanoma stage I and II who were treated with SLN biopsy between 2001 and 2007.

**Results:**

Micrometastases in inguinal SLN were detected in 32 patients, 14 were single, 2 were double, and 16 were multiple. Twenty-six patients had macrometastases.

**Conclusions:**

No patient with any micrometastases or a single SLN macrometastasis in the inguinal region had any iliac/obturator non-sentinel metastases after CLND in our series. Furthermore, no patient with single SLN micrometastasis in the inguinal region had any non-sentinel metastases at all after CLND in our series. In these cases respective CLND might be omitted.

## Introduction

Since its introduction by Morton in 1992, sentinel lymph node biopsy (SLNB) is now becoming a standard of care for staging of patients with clinical stage I and II cutaneous melanoma or breast cancer.^1^–^3^ Sentinel lymph node (SLN) predicts the status of regional nodal basin and its surgical recovery nears 100%.^4^ The standard of treatment for positive SLN is completion lymph node dissection (CLND).^5^ Specifically, in the inguinal region the extent of CLND is not yet clearly defined. Both inguinal and inguino-iliac/obturator lymph node dissection are being performed.

Before the introduction of SLNB the standard of treatment for palpable metastatic inguinal lymph nodes in patients with cutaneous melanoma was inguino-iliac/obturator lymph node dissection. This was due to the fact that up to 40% of patients with palpable metastatic inguinal lymph nodes had metastases in the iliac or obturator nodes.^6^

Nowadays, the situation has changed. The metastatic deposits found in the SLN are much smaller than previously detected palpable metastases and thus the chance of iliac or obturator node metastasis is considerably lower. Frequently, the CLND after positive inguinal SLN is completely negative thus adding the patient only additional complications and morbidity, and no benefit.^4^,^5^,^7^ Namely, only 10–30% of patients with SLN metastases in any region have additional metastases in non-sentinel lymph nodes.^8^ There are even fewer patients with iliac or obturator metastases after positive inguinal SLN.^9^ Therefore, inguinal dissection might be radical enough procedure after positive inguinal SLN. However, at present time, it is difficult to predict in which subset of inguinal SLN positive patients the iliac/obturator lymph node dissection would not present with any further positive lymph nodes. Through the literature review we did not find any author who dealt specifically with the pattern of non-sentinel metastasis after a positive inguinal SLN.

The aim of our study was to determine the predictive value of inguinal SLN micrometastases subdivided according to the number of micrometastases in predicting non-sentinel inguinal, iliac or obturator lymph node involvement in CLND. In addition, we reviewed files of melanoma patients who were surgically treated for palpable inguinal metastases in the same time period.

## Patients and methods

### Patients

The files of all 743 consecutive and prospectively enrolled patients with primary cutaneous melanoma stage I and II who were treated with SLNB at the Institute of Oncology, Ljubljana, Slovenia between January 2001 and December 2007 were reviewed retrospectively in our study. Positive inguinal SLN was detected in 58 patients (32 female, 26 male, median age 55 years). In addition, we reviewed files of 94 stage III melanoma patients who were surgically treated for palpable inguinal metastases at our department in the same time period.

The investigators strictly followed recommendations of the Helsinki declaration and of the European Council Convention on Protection of Human Rights in Bio-Medicine.

### Methods

Preoperatively, ultrasound of the inguinal region was performed in most patients. Criteria for SLNB were as follows: cutaneous melanoma stage I or II thicker than 1 mm or thinner than 1 mm, but with signs of gross regression, ulceration or Clark level IV/V. SLNB and wide local excision were performed using the standard triple technique of preoperative lymphoscintigraphy using ^99m^Tc radiolabelled nano-colloid, followed by perioperative intradermal injection of Patent blue dye and intraoperative use of a hand-held sterile gamma probe.^10^ SLN was defined as the only hot and/or blue node, the hot and/or blue node receiving afferent lymphatic channel from the tumour and the hot and/or blue node which was the first one in sequential pattern.

The SLN was examined histopatologically by using routine HE and immunohistochemical staining for S-100 protein and HMB-45 antigen as previously reported.^10^ Briefly, SLN were bisected, fixed in formalin and embedded in paraffin. A maximum of 8 sections of each half were made, odd sections were stained with routine HE and even sections were immunohistochemically stained for S-100 except for the section 6 which was immunohistochemically stained for HMB45. For the purpose of our study the definitions of micrometastases and macrometastases are metastatic deposits within SLN smaller or equal to (≤) 2 mm and larger than (>) 2 mm, respectively. We acknowledge that this is in contrast to the current TNM classification where micrometastases and macrometastases are defined as clinically non palpable and palpable metastatic nodes respectively.^11^ A cluster of cells (10 - 30 grouped cells) or isolated tumour cells (up to 20 individual cells) were also defined as micrometastasis.^12^ The number of micrometastases and macrometastases was also recorded.

Every patient with metastatic inguinal LN underwent a (C)LND of the involved nodal basin regardless of the metastasis size (palpable or non-palpable, identified at the SLNB) or number. Inguinal or inguino-iliac/obturator lymph node dissection was performed depending on surgeon’s personal decision, guided mostly by the age and performance status of the patient. The metastatic involvement of non-sentinel inguinal, iliac or obturator lymph nodes was recorded. The nodes in the CLND specimens were evaluated by the routine HE technique. Adjuvant radiotherapy was given to the patients with more than 3 cm metastatic nodes, with more than 3 metastatic nodes or with metastatic extra capsular extension.

### Statistical analysis

Numerical variables are presented by its mean, median, followed by minimum and maximum values. Attributive data are described as the absolute numbers with corresponding relative frequencies. The overall survival rates were analysed using Kaplan-Meier method.^13^ Log-rank test was used to test the equality of the overall survival curves from the time of diagnosis until death of any cause. Chi-squared test was used to determine the statistical significance of the association between inguinal SLN histology and CLND status. All tests were two-sided. A *p* value of less than 0.05 was considered statistically significant. The statistical analysis was performed using the SPSS/PC software package (version 13.0. for Windows).

## Results

### Inguinal metastases detected after SLNB

Between January 2001 and December 2007, all lymphatic basins SLNB were performed in 743 patients. Overall, positive inguinal SLN was detected in 58 cases. Median follow-up of these SLN positive patients was 1.87 years (range 0.76–7.17). Clinicopathological characteristics of the patients with positive inguinal SLN are given in Table 1 and additional data for 93 patients with palpable inguinal metastases are given in Table 2.

Twenty-six (44.8%) patients had macrometastases in the inguinal SLN. Micrometastases (including clusters of cells and isolated tumour cells) in inguinal SLN were detected in 32 (55.2 %) patients.

The patients with micrometastases were divided into three groups on the basis of the micrometastases number in the inguinal SLN: there were 14 patients with single micrometastasis, 2 patients with double micrometastases in one node, and 16 patients with more than two micrometastases in one or more lymph nodes (Table 1). Inguinal or inguino-iliac/obturator CLND was performed in all patients depending on surgeon’s personal decision, guided mostly by the age and performance status of the patient.

Out of 14 patients with solitary and 2 patients with double micrometastases, all were without additional metastases in non-sentinel lymph nodes regardless of the type of CLND performed. Out of 16 patients with 3 or more micrometastases, only 1 had additional positive non-sentinel lymph nodes. Altogether, there were 11 patients with metastases in non-sentinel lymph nodes; 1 had multiple micrometastases in the inguinal SLN and 10 had macrometastases in the inguinal SLN. The difference between CLND negative and positive groups of patients, divided according to inguinal SLN tumour burden, was statistically significant with Chi-square *p* value of 0.01 (Table 3).

Inguinal CLND was performed in 40/58 (69%) patients. The median follow up in this group was 1.59 years (range 0.76–5.69). Seven patients had positive non-sentinel nodes (5 patients had 1 positive non-sentinel node, 1 patient had 2 and 1 patient had 4 positive non-sentinel nodes). Out of those 7 patients, 6 were disease free upon follow up and 1 died with gross melanosis of the leg without systemic progress.

Inguino-iliac/obturator CLND was performed in 18/58 (31%) patients. The median follow up of these patients was 2.71 years (range 0.83–7.17). Four patients had positive non-sentinel nodes (3 patients had 1 positive non-sentinel node and 1 patient had 5 positive non-sentinel nodes). Out of those 4 patients, 3 were disease free upon follow up and 1 died from systemic progress.

Adjuvant postoperative radiotherapy was given to 2 patients (3.5%). Additional 17 patients (29.3%) received palliative radiotherapy later in time due to disease progression.

**FIGURE 1:**
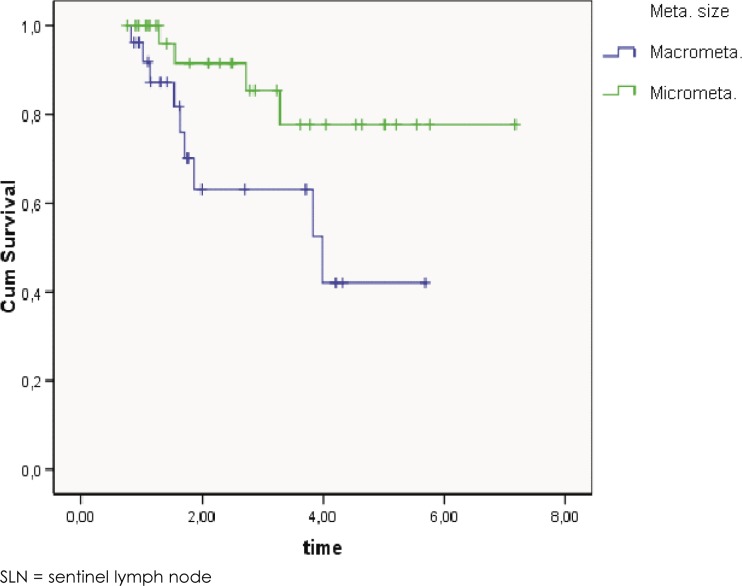
The log rank test of Kaplan-Meier overall survival curves for patients with SLN micrometastases compared to patients with SLN macrometastases (*p* = 0.032).

Not surprisingly, the log rank test of Kaplan-Meier survival curves showed a statistically significant better survival (Figure 1, *p* = 0.032) for patients with SLN micrometastases (91.5% overall survival at 2 years, CI 84.1 % - 98.9%, median follow up 2.5 years) compared to patients with SLN macrometastases (64.0% overall survival at 2 years, CI 50.3% - 77.7%, median follow up 1.6 years).

On the other hand, there was no statistical difference after log rank test of Kaplan-Meier survival curves (*p* = 0.604) for patients after inguinal CLND compared to patients after inguino-iliac/obturator CLND.

### Palpable inguinal metastases

In addition, there were 93 stage III melanoma patients who were surgically treated for palpable inguinal metastasis in the same time period. Inguinal LND was performed in 21/93 (23%) patients while inguino-iliac/obturator LND was performed in 72/93 (77%). There was not any statistical difference in the log rank test of Kaplan-Meier survival curves (*p* = 0.420) between patients when comparing the type of dissection performed after palpable inguinal metastases.

On average, there were 3.45 positive LN after palpable inguinal metastases while there were only 1.28 positive LN after positive inguinal SLNB (Tables 1 and 2). There were 21/93 (22.6%) patients with positive iliac/obturator LN after palpable inguinal metastases while there were only 3/58 (5.2%) patients with positive iliac/obturator LN after positive inguinal SLNB.

**FIGURE 2:**
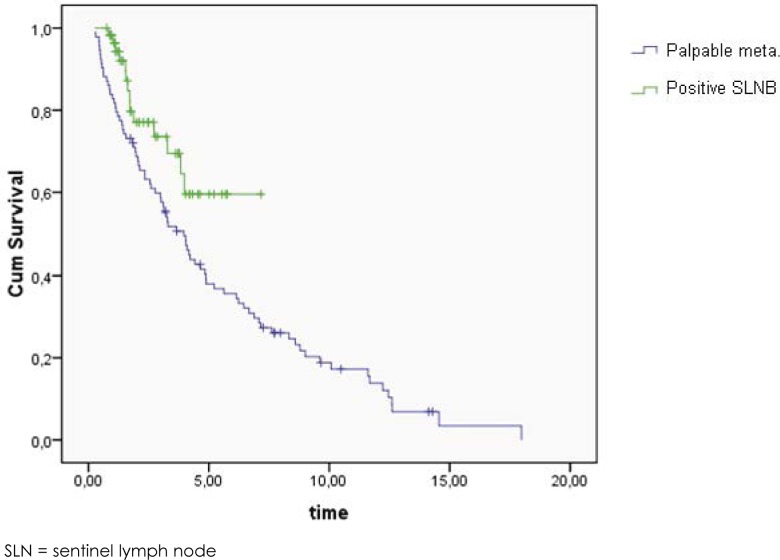
The log rank test of Kaplan-Meier overall survival curves for patients after positive inguinal SLN compared to patients after palpable inguinal metastases (*p* = 0.028).

The log rank test of Kaplan-Meier survival curves showed a statistically significant better overall survival (Figure 2, *p* = 0.028) for patients with positive inguinal SLNB (77.1% survival at 2 years, CI 64.4% - 89.8%, median follow up 1.9 years) than for patients with palpable inguinal metastases (70.5% survival at 2 years, CI 60.3% - 807%, median follow up 3.3 years).

## Discussion

As we have shown in our previous study, metastases in non-sentinel lymph nodes in patients with micrometastases in SLN are a rare event regardless of the lymphatic region. In fact, no patient with a single SLN micrometastasis in any region had metastases in CLND.^7^

Our study focused on the rates of inguinal, iliac and obturator non-sentinel metastatic involvement in relation to micrometastases, macrometastases and number of micrometastases in the inguinal SLN.

Due to CLND associated morbidity, such as scarring, limb oedema, seroma formation, paresthesias and deterioration of pre-existing medical co-morbidities, identification of patients without non-sentinel lymph node involvement would be of great clinical importance. Specifically in the inguinal region, the pattern of non-sentinel lymph node involvement of inguinal, iliac and obturator nodes is of great interest, due to possibility of omitting the iliac/obturator part of the CLND.

We observed that more than half (55.2%) of the positive SLN were micrometastatic, which presents a rather high percentage, yet similar to the results reported by other authors.^14^ This fact might be explained by the selection of patients because the majority of our patients underwent preoperative ultrasound of the inguinal region, thus detecting at least some larger metastases that are otherwise too small to be palpated.

In 14 cases, SLN micrometastases were single, while in 2 they were double, and in 16 multiple. We observed that no patient with any micrometastases or a single SLN macrometastasis had any iliac/obturator non-sentinel metastases after CLND. Furthermore, no patient with single SLN micrometastasis had any non-sentinel metastases at all after CLND. Statistically, the difference in finding additional positive nodes after CLND between the groups with inguinal SLN micrometastases and macrometastases was statistically significant (*p* = 0.01) confirming the fact that the obvious difference between the mentioned groups is not a coincidence.

Iliac/obturator CLND might be avoided after detecting a single macrometastasis or any micrometastases in the inguinal SLN and CLND of any type might be avoided after detecting a single micrometastasis in the inguinal SLN. These statements are additionally supported (although clearly not proven) by the absence of statistically significant survival difference between Kaplan-Meier survival curves (*p* = 0.604) for patients after inguinal CLND compared to patients after inguino-iliac/obturator CLND in the SLNB positive patients and even in patients with palpable inguinal melanoma metastases. Another fact supporting our proposal for omitting the iliac/obturator part of CLND after micrometastases in SLN is that out of 7 patients with additional metastases after only inguinal CLND, 6 were disease free upon last follow up and 1 died with gross melanosis of the leg without systemic progress.

On the other hand, we found iliac/obturator metastases in 21% of patients with palpable inguinal melanoma metastases and there was statistical difference in survival between patients with positive inguinal SLNB and palpable inguinal metastases (Figure 2). Hence, full inguino-iliac/obturator LND is still recommended after finding palpable inguinal metastases.

One of the first researchers to deal with the subject of non-sentinel metastases was Starz *et al.* in 2001.^15^ His group tried to predict non-sentinel node involvement by creating so called “S classification” using two morphometric parameters: the number of tumour-involved, 1-mm slices of the SLN and the centripetal depth of metastatic cell invasion. Their otherwise quite complicated system of classification has shown that there are no further metastases when SLN metastasis is less or equal to 1 mm and located peripherally. The Eggermont study revealed a group of patients with sub-micrometastases (< 0.1 mm) that had no non-sentinel metastases and are thus unlikely to benefit from CLND.^16^ Similarly, other authors are trying to determine which SLN characteristics prognosticate no further metastases to non-sentinel lymph nodes (Table 4). Vuylsteke *et al.* found this to be primary cutaneous melanoma with Breslow thickness of 2.5 mm or less and the surface of metastases in SLN of 0.3 mm^2^ or less.^19^ Dewar *et al.* described it as only subcapsular localisation of metastases in SLN^18^ and Cochran *et al.* reported it as a relative metastases surface to SLN surface of 1% or less.^17^ In contrast, Carlson *et al.* were unable to predict no involvement to non-sentinel lymph nodes by any known parameter.^14^ The difference between Carlson’s study and ours is that we subdivided micrometastases to single, double or multiple micrometastases that yielded the group of single micrometastasis that had no additional metastases in non-sentinel lymph nodes. Through the literature review we did not find any author who dealt specifically with the pattern of non-sentinel metastasis after a positive inguinal SLN.

At present, we feel that, after micrometastases in the inguinal SLN are detected, iliac/obturator CLND can be omitted. However, this question needs to be addressed in a properly designed prospective trial.

## Figures and Tables

**TABLE 1:** Patients’ clinicopathological characteristics after positive inguinal SLNB

**Characteristic**	**SLNB group**
Total, n	58
Sex, n (%)	
Female	32 (55.2%)
Male	26 (44.8%)
Age, years	
Median	55
Range	7.6–87.1
Primary site, n (%)	
Trunk	11 (19%)
Lower extremity	47 (81%)
Unknown	0 (0%)
Breslow, mm	
Mean	3.66
Median	3.2
Range	1.1–13
0–1, n (%)	0 (0%)
1.01–2, n (%)	14 (24.1%)
2.01–4, n (%)	30 (51.8%)
>4.01, n (%)	14 (24.1%)
Unknown	0 (0%)
Clark, n (%)	
III	11 (19%)
IV	36 (62%)
V	4 (6.9%)
Unknown	7 (12.1%)
Ulceration, n (%)	
Yes	30 (51.7%)
No	21 (36.2%)
Unknown	7 (12.1%)
SLN removed, n	
Mean	1.86
Range	1–4
SLN status, n (%)	
1 micrometastasis	14 (24.1%)
2 micrometastases	2 (3.5%)
2+ micrometastases	16 (27.6%)
Macrometastases	26 (44.8%)
CLND nodes removed, n (mean)	
Inguinal dissection	10.1
Inguino-iliac dissection	19.3

SLNB = sentinel lymph node biopsy; SLN = sentinel lymph node; CLND = completion lymph node dissection

**TABLE 2:** Patients’ clinicopathological characteristics after palpable inguinal metastases

**Characteristic**	**Palpable metastases**
Total, n	93
Sex, n (%)	
Female	53 (57%)
Male	40 (43%)
Age, years	
Median	66
Range	18–75
Primary site, n (%)	
Trunk	9 (10%)
Lower extremity	71 (76%)
Unknown	13 (14%)
Breslow, mm	
Mean	4.58
Median	3.45
Range	0.5 - 25
0–1, n (%)	7 (7%)
1.01–2, n (%)	12 (13%)
2.01–4, n (%)	22 (24%)
>4.01, n (%)	26 (28%)
Unknown	26 (28%)
Clark, n (%)	
III	14 (15%)
IV	34 (37%)
V	7 (7%)
Unknown	33 (36%)
Ulceration, n (%)	
Yes	26 (28%)
No	9 (10%)
Unknown	58 (62%)
Palpable nodes positive, n	
Mean	3.45
Range	2–26
LND nodes removed, n (mean)	
Inguinal dissection	12.9
Inguino-iliac dissection	16.7

LND = lymph node dissection

**TABLE 3:** Association between CLND negative and positive patients divided according to micrometastases and macrometastases

	**CLND negative**	**CLND positive**	**Total**	
micrometastases	31	1	32
macrometastases	16	10	26
Total	47	11	58

Chi-square, *p* = 0.01; CLND = completion lymph node dissection

**TABLE 4:** Studies reporting on SLN characteristics predictive for the absence of additional metastases in non-sentinel lymph nodes

**Author**	**N**	**SLN characteristic(s)**
Carlson *et al.* 2003^14^	104	Not found
Cochran *et al.* 2004^17^	90	Tumour area <1%
Dewar *et al.* 2004^18^	146	Subcapsular location
Vuylsteke *et al.* 2005^19^	71	Breslow <2.5mm, tumour load <0.3mm^2^
van Akkooi *et al.* 2006^16^	77	Micrometastases <0.1mm
Glumac *et al*. 2008^7^	74	Single micrometastasis <2mm

N = Number of patients with positive SLN; SLN = sentinel lymph node
